# Mixed outcomes for computational predictions

**DOI:** 10.7554/eLife.22661

**Published:** 2017-01-19

**Authors:** Chi Van Dang

**Affiliations:** Abramson Cancer Center, Abramson Family Cancer Research Institute, University of Pennsylvania, Philadelphia, United Statesdangvchi@upenn.edu

**Keywords:** Reproducibility Project: Cancer Biology, replication, metascience, reproducibility, drug retargeting, cimetidine, Human

## Abstract

Experimental efforts to validate the output of a computational model that predicts new uses for existing drugs highlights the inherently complex nature of cancer biology.

**Related research articles** Kandela I, Aird F, Reproducibility Project: Cancer Biology. 2017. Replication Study: Discovery and preclinical validation of drug indications using compendia of public gene expression data. *eLife* **6**:e17044. doi: 10.7554/eLife.17044

Kandela I, Zervantonakis I, Reproducibility Project: Cancer Biology. 2015. Registered Report: Discovery and preclinical validation of drug indications using compendia of public gene expression data. *eLife* **4**:e06849. doi: 10.7554/eLife.06849

In 2011 researchers at Stanford and the Lucile Packard Children's Hospital reported a computational approach for predicting if a drug that was currently approved for the treatment of a certain disease could also be used to treat a form of cancer (Sirota et al., 2011). The researchers derived gene expression signatures for 100 diseases from data available in the Gene Expression Omnibus (GEO) and compared these signatures with gene expression measurements on 164 drugs.

Based on these comparisons Sirota et al. predicted that cimetidine, an antiulcer drug, could be used to treat a form of lung cancer called lung adenocarcinoma, and then went on to demonstrate the efficacy of cimetidine against this form of cancer both in vitro and in vivo (in xenograft experiments in which human tumor cells were transplanted into mice). They also confirmed, as predicted by their computational model, that cimetidine was not effective against renal carcinoma.

In 2015, as part of the Reproducibility Project: Cancer Biology, Kandela et al. published a Registered Report (Kandela et al., 2015) which explained in detail how they would seek to replicate selected experiments from Sirota et al. The results of these experiments have now been published as a Replication Study (Kandela et al., 2017).

In the original work, Sirota et al. demonstrated that cimetidine induced the death of the lung cancer cell line A549. To corroborate this finding, they tested three doses of cimetidine against A549 tumors that had been implanted in mice. While tumors treated with a negative control grew to 3.25 times their original volume, and tumors treated with a positive control (an established cancer drug called doxorubicin) doubled in size, tumors treated with the highest dose of cimetidine grew to 2.3 times their original volume, which was statistically significant. However, xenograft studies are inherently complex – there is a lot of variability in the length of time it takes a tumor to become established after implantation, and tumors also grow at different rates in different animals – and the effects of doxorubicin and cimetidine on the growth rates of tumors do not seem very robust from a biological point of view.

In the Replication Study, Kandela et al. found that cimetidine treatment in the lung adenocarcinoma xenograft model resulted in decreased tumor sizes compared to a negative control treatment (Kandela et al., 2017). However, while the effects were in the same direction as those reported by Sirota et al., they were not significant when a Bonferroni correction was used to adjust for multiple comparisons. Treatment with doxorubicin also reduced the size of the lung tumors compared to a control, but again the effects were not significant. In both cases, however, a statistically significant effect was observed when the dataset from the original paper and the dataset from the Replication Study were combined in a meta-analysis.

The findings of the Replication Study raise issues related to robustness, statistical methods, and effect sizes. Robustness characterizes the consistent response of a system to perturbations: the more robust the system, the less influence these perturbations have on its output. There are many factors that could influence the robustness of the xenograft models used in these experiments: batch effects on the efficacy of the drugs used; changes in the properties of cell lines over time; the strains of the mice used, and also their sex; factors related to microbiome and chow; circadian effects; temperature; and the antimicrobials that might be used in certain facilities.

It is also possible that Sirota et al. paid too much attention to statistical significance and P values and not enough attention to the actual size of the effect being investigated (Motulsky, 2014). Indeed, the actual tumor sizes observed by Sirota et al. were not diminished by cimetidine at early time points, and the reduction in tumor size at the last time point (the only time point at which the reduction was statistically significant) was only about 30%. This moderate effect size might have contributed to the fact that the effects seen in the replication were similar to those in the original experiments, but not statistically significant (although, as mentioned above, combining data from the two studies did give significant results).

There have been growing concerns about irreproducibility in the biological literature in recent years (Begley and Ellis, 2012; Errington et al., 2014; Baker, 2016). A powerful lesson to emerge from this Replication Study is that reproducibility is not black and white because, like many areas of research, cancer biology is nuanced and inherently complex.

## Note

Chi Van Dang was the eLife Reviewing Editor for the Registered Report (Kandela et al., 2015) and the Replication Study (Kandela et al., 2017).
